# Damage Quantitative Detection of Curved Composite Laminates Based on Improved Particle Swarm Optimization Algorithm

**DOI:** 10.3390/ma18102317

**Published:** 2025-05-16

**Authors:** Shuxia Tian, Shunqiang Wang, Zhenmao Chen, Ran Hao, Zhihui Qin, Jiangdong Ma, Linfeng Xu

**Affiliations:** 1 Henan Key Laboratory of Intelligent Manufacturing of Mechanical Equipment, Zhengzhou University of Light Industry, Zhengzhou 450002, China; tiansx@zzuli.edu.cn (S.T.); 3323040400425@zzuli.edu.cn (S.W.); 332102040141@zzuli.edu.cn (R.H.); 331902040103@zzuli.edu.cn (Z.Q.); 332202040186@zzuli.edu.cn (L.X.); 2 State Key Laboratory for Strength and Vibration of Mechanical Structures, Xi’an Jiaotong University, Xi’an 710049, China; 3 Henan Boiler and Pressure Vessel Inspection Technology Research Institute, Zhengzhou 450016, China; majiangdong123@126.com

**Keywords:** curved fiber-reinforced composites, structural damage assessment, finite difference method, swarm intelligence optimization, damage quantification

## Abstract

In order to solve the problem of damage identification of composite laminates during processing and service, a quantitative damage detection method based on swarm intelligence optimization was proposed for structural damage detection of curved composite laminates. Firstly, the structural damage element was defined by the method of reducing the elastic modulus of the element, and the modal parameters of the numerical model of the laminate under different damage conditions were obtained by analyzing the structural vibration characteristics. Secondly, the objective function was constructed from the vibration data, and the precise location and degree of damage were quantitatively calculated by the swarm intelligence optimization algorithm. In order to prevent the particles from falling into the local optimal, the boundary rebound strategy was used to process the boundary, and the MS operator was introduced to greatly accelerate the convergence speed of the algorithm. The numerical results indicate that without the influence of noise, the algorithm was not affected by the quantity, location or size of the damage and could effectively detect damage in curved fiber-reinforced composites, with the detection error rates being within 0.5%. After adding 1% and 5% noise to the frequency and vibration mode, respectively, the convergence speed of the algorithm slowed down, and the convergence times obviously increased. However, it could still accurately locate the damage, and the calculation error of the damage degree was less than 6%. Finally, the effectiveness of the proposed algorithm was verified through experimental tests.

## 1. Introduction

Composite materials have a series of advantages such as low relative density, high specific strength and stiffness, impact resistance, rapid heat dissipation and insulation, and they have been widely used in many fields such as aerospace, automotive, infrastructure and pipelines [1,2]. Due to the complexity and instability of the manufacturing process of carbon fiber composite materials, as well as the complex stress environment during service, layered composite structures inevitably suffer from damage such as debonding, delamination and fracture. Among these, interlayer delamination damage is the most common and main form of damage. When a structure experiences delamination damage, its overall strength decreases, which in turn affects the safety of the component during service [3,4]. Therefore, it is necessary to develop a reliable and efficient structural damage assessment method for layered composite structures.

In recent years, more and more damage detection methods have emerged, especially for large structural components [5,6,7]. Dynamic damage detection methods have shown great advantages [8,9]. However, many methods currently suffer from sensitivity and accuracy issues in damage detection or have unpredictable probabilities of false or missed detections, thereby reducing the reliability of the methods. At the same time, existing dynamic damage detection methods mostly focus on simple planar structures or spatial truss structures. Their common feature is that the topology structure is simple, and the subsequent detection of dynamic signals and extraction of damage information are also relatively easy to achieve [10,11]. However, for most aerospace vehicles, high-speed trains, etc., composite materials are mostly applied to surface configurations with complex topology structures. Existing damage detection methods often lack universality, which is also the limitation of current damage detection methods based on structural dynamic information in complex structures. Therefore, it is of great significance to seek an effective and accurate dynamic damage detection method to solve the quantitative analysis and calculation problems of damage in the preparation and service process of layered composite structures with curved configurations.

In order to accurately predict the degree of damage to complex structures, many researchers have proposed various metaheuristic optimization algorithms in the past few years, which are known for their potential in dealing with complex nonlinear engineering problems. Mahdavi et al. [12] proposed a framework structure structural damage assessment strategy based on two-step vibration by combining an evolutionary optimization algorithm and the supervised machine learning paradigm. Guedria [13] proposed an accelerated differential evolution algorithm with a new operator for multi-damage detection in plate-like structures. Banimahd et al. [14] proposed a damage detection method using the artificial bee colony algorithm, which detects structural damage to beams through modal and frequency analysis. However, this method still has certain limitations in damage detection for composite structures, and the accuracy of the algorithm results and how to reduce the large amount of computational costs are issues that need to be considered.

Particle swarm optimization (PSO) is a novel global optimization technique based on swarm optimization. Due to its simplicity, wide applicability, and excellent computational performance, it has received widespread attention. In addition to theoretical research, it has also been used to solve various practical optimization problems [15,16]. The improved particle swarm optimization (IPSO) algorithm proposed in this paper has higher computational efficiency compared with optimization algorithms such as particle swarm optimization (PSO) and the grey wolf optimizer (GWO), and it achieves better optimization effects for function optimization and engineering problems.

Therefore, based on the structural vibration response parameters, this article applies the IPSO algorithm to the damage detection of layered composite structures with curved configurations. Meanwhile, in order to improve the convergence speed and accuracy, a two-stage damage quantitative detection method based on the differential method and IPSO algorithm is proposed. Firstly, based on the finite difference method, the approximate area of damage can be identified, and a set of potential damage units can be found to reduce the number of variables and computation time in the optimization problem. Secondly, the IPSO algorithm was used to calculate the precise location and degree of damage. In addition, this article also studies the effects of minor damage, major damage and noise on the accuracy of the proposed algorithm. The numerical calculation results show that this method can reduce computational costs and effectively and quantitatively analyze the location and degree of damage.

## 2. Mathematical Model of Damage Detection Method Based on Vibration Response Parameters

### 2.1. Parameterization of Damage

In this paper, the finite element method (FEM) was used to discretize the composite laminates and perform modal analysis to calculate their vibration characteristics. The generalized characteristic equation of the free vibration of the structures with N degrees of freedom is as follows:

(1) $$\left(K-\omega_{i}^{2}M\right)\psi_{i}^{2}=0,i=1,2,\ldots ,n,$$

where *K* and *M* are the stiffness matrix and mass matrix of the structure, respectively, and $\omega_{i}$ and $\psi_{i}$ are the natural frequencies and mode shapes of the *i*th order, respectively.

During the modeling process, the damage can be set by reducing the stiffness characteristics of the structure (such as the elastic modulus, cross-sectional area or moment of inertia) while ignoring the impact of damage on the mass. In this paper, the damage of composite laminates is simulated by reducing the elastic modulus. Then, the damage degree of the damaged element can be expressed as follows:

(2) $$x_{e}=\frac{E-E_{d}}{E},e=1,\ldots ,N,$$

where *E* represents the elastic modulus of the healthy unit, $E_{d}$ represents the elastic modulus of the damaged unit and $x_{e}\in \left(0,1\right)$, where 0 represents that the unit is healthy and 1 represents the unit being completely damaged.

Based on the finite element method, when the damage model is established to calculate the structural vibration characteristics, the overall stiffness matrix is composed of the damaged element’s stiffness matrix and the healthy element’s stiffness matrix:

(3) $$K=\sum_{e=1}^{N}\left(1-x_{e}\right)k_{e},$$

where *N* represents the total number of elements after plate dispersion and $k_{e}$ represents the element’s stiffness matrix.

### 2.2. Damage Location Recognition Algorithm Based on Finite Difference Method

The modal parameters of the structure, such as the frequency, mode and damping ratio, are related to the structural damage. Therefore, the changes in these modal parameters between the damaged and healthy structures can be used to locate the damage. In addition, the flexible matrix, mode curvature and modal strain energy established by these modal parameters are also considered to be effective indicators of damage. In previous studies [17], the authors proved that the finite difference method has certain advantages in locating damage.

For the plate structure, based on the central finite difference method, the vibration mode curvature in both directions of the measuring point (i,j) can be expressed as follows:

(4) $$\left\{\begin{array}{c} u_{xx}=\frac{\mu \left(x_{i+1,}y_{j}\right)-2\mu \left(x_{i}y_{j}\right)+\mu \left(x_{i-1,}y_{j}\right)}{h_{x}^{2}}\\ u_{yy}=\frac{\mu \left(x_{i,}y_{j+1}\right)-2\mu \left(x_{i,}y_{j}\right)+\mu \left(x_{i,}y_{j-1}\right)}{h_{y}^{2}} \end{array}\right.,$$

where μ represents the node displacement of the measuring point (i,j) and *h* represents the distance between two adjacent measuring points.

According to Equation (4), the modal curvatures before and after damage are calculated, and then the modal curvature before and after the damage is subtracted to establish the structural damage assessment index as follows:

(5) $$d\left(x_{i},y_{j}\right)=\left[\left|u_{xx}^{D}\left(x_{i},y_{j}\right)-u_{xx}\left(x_{i},y_{j}\right)\right|+\right.{\left.\left|u_{yy}^{D}\left(x_{i},y_{j}\right)-u_{yy}\left(x_{i},y_{j}\right)\right|\right]}^{2},$$

where $u_{xx}^{D}\left(x_{i},y_{j}\right)$ and $u_{xx}\left(x_{i},y_{j}\right)$ are the curvature of the vibration modes of the damaged and undamaged structures in the *x* direction, respectively. By analyzing the above structural damage assessment indexes, it can be seen that if the structure to be tested is an undamaged laminated plate, and the difference between the two groups of data is noise, then the damage index *d* is plotted as the *z* direction. The peak value only fluctuates slightly near zero. On the contrary, if the structure to be measured is a damaged laminate, then there will be a large peak value at the damaged area. This can be used to determine the damage condition of the laminates.

### 2.3. Objective Function of Optimization Algorithm Based on Vibration Response Parameters

Changes in structural characteristics can lead to changes in the vibration frequency and mode, which is the driving force for using modal methods for structural damage assessment and health monitoring. The damage assessment process is usually achieved by minimizing the modal parameters of the tested structure and iteratively updating the modal parameters of the finite element model to construct the objective function. Once the damage occurs, the stiffness of the structure will decrease, and then the flexibility will increase accordingly. Therefore, the objective function can be established by calculating the difference between the flexibility matrix values of the tested structure and the finite element model:

(6) $$f\left(x\right)=\lambda \left(x\right)\left(\frac{{∥F^{E}-F^{A}\left(x\right)∥}^{2}}{{∥F^{E}∥}^{2}}\right),$$

where *F* is the flexibility matrix of the structure. Superscripts *E* and *A* represent the data of the damaged structural under testing and the finite element model data, respectively, while ∥o∥ represents the Frobenius norm of the matrix, and $x=\left(x_{1},\ldots ,x_{N}\right)\in {\left[0,1\right]}^{N}$ is the damage degree vector of the identified damage unit. The penalty function λ(x) can be expressed as follows:

(7) $$\lambda \left(x\right)=\left(1+\frac{M(x)}{N}\right),$$

In this formula, M(x) represents the number of damaged units in the identified damage area, and *N* represents the total number of units in the identified damage area. Integrating the penalty function into the objective function not only minimizes the calculated values between the experimental flexibility matrix and the numerical model flexibility matrix, but also avoids misjudgment of healthy units.

Therefore, the structural damage assessment optimization problem can be expressed as minf(x), where $0\leq x_{i}\leq 1.\;i=1,\ldots ,N$. By continuously iterating the degree of damage of *N* elements, the defined objective function f(x) can be minimized to achieve quantitative identification of damage.

### 2.4. Damage Quantitative Identification Algorithm Based on IPSO

#### 2.4.1. Standard PSO Algorithm

**Figure 1:**
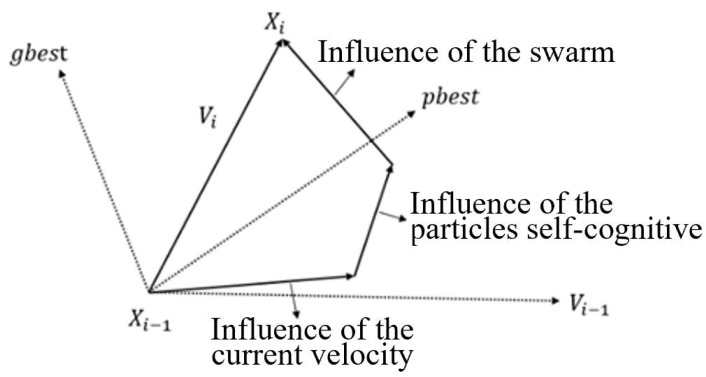
Schematic diagram of particle position updating.

PSO is a kind of swarm intelligence optimization algorithm that simulates the foraging activities of birds [18]. Each particle has two attributes: speed and position. According to the swarm optimal solution found by each particle and the optimal solution found by the current particle, it constantly adjusts its speed and position to search for the optimal solution to the objective function. The schematic diagram of particle updating is shown in Figure 1:

Assuming that there are n particles in a population, $x_{i}$ and $v_{i}$ present the position and speed of each particle at time *i*, respectively, pbest represents the optimal solution for the current particle in the search history, and gbest represents the optimal solution of the whole particle swarm at the current time. The update formula of the particle’s speed and position at time *i* in the algorithm is

(8) $$\left\{\begin{array}{c} V_{i}=V_{i-1}\times w+C1\times \;\mathrm{rand}\;1\times \left(\;\mathrm{pbest}\;-X_{i-1}\right)+\\ C2\times \;\mathrm{rand}\;2\times \left(\;\mathrm{gbest}\;-X_{i-1}\right)\\ X_{i}=X_{i-1}+V_{i} \end{array}\right.,$$

where *w* is the inertia weight of the speed, C1 is the self-learning factor, C2 is the social learning factor and rand1 and rand2 are random numbers generated in the range [0,1], $X_{i}$.

#### 2.4.2. Improved PSO Algorithm

PSO has the advantages of requiring fewer parameters and a faster optimization speed, but at the same time, standard PSO lacks diversity of its own particles and can easily fall into local convergence. Therefore, according to the needs of practical problems in this paper, the following improvements have been made to standard PSO to increase the diversity of solutions in order to jump out of local convergence and find the global optimal solution.

(1) Improving the inertia weight and learning factor

In the initial stage of iteration, the algorithm should pay attention to global search, emphasize the self-cognitive ability of particles, pay attention to the ergodicity of particles, and reduce the probability of falling into local convergence for the optimal solution. With the progress of iteration, in order to strengthen the communication between particles and make the position of the optimal solution for the population have a greater impact on the operation of each particle, more attention should be paid to the local search of the optimal solution in the later stage of iteration to improve the accuracy of the solution. At the same time, a larger inertia weight is beneficial to the global search, and a smaller inertia weight is beneficial to the local search. Therefore, in order to achieve the best balance between global search and local search, the inertia weight and learning factor set a linear decreasing or increasing search strategy. The inertia weight *w*, the selflearning factor C1 and the social learning factor C2 are set as follows:

(9) $$\left\{\begin{array}{c} w=w_{\max}-i/ter\times \left(w_{\max}-w_{\min}\right)\\ C1=C_{\max}-i/ter\times \left(C_{\max}-C_{\min}\right),\\ C2=C_{\min}-i/ter\times \left(C_{\min}-C_{\max}\right) \end{array}\right.$$

where *i* is the current iteration number, ter is the maximum iteration number and $w_{\max}$ and $w_{\min}$ are the maximum and minimum values for the inertia weight, respectively. In this paper, $w_{\max}=0.95,\;w_{\min}=0.4,\;C_{\max}$ and $C_{\min}$ are the maximum and minimum values for the learning factors, respectively, and $C_{\max}=2.5$ and $C_{\min}=1.5$ are taken.

(2) MS operator

The MS operator is often introduced into PSO to speed up the convergence of the algorithm. The basic idea is that after the laminated plate is discretized into many units, the number of healthy units in the structure is far greater than the number of damaged units. By using the MS operator, a unit with an extremely low damage degree is regarded as a healthy unit so as to speed up the movement of each variable of the particles and reduce the influence of non-damage factors on the health degree of a truly damaged unit. If a healthy unit is judged to be a weakly damaged unit, then the damage degree of the truly damaged unit is bound to be smaller. Therefore, in the iterative process of this article, units with damage levels less than 10% after every five iterations are set as healthy units.

(3) Boundary rebound strategy

In order to prevent particles from falling into the local optimum in the process of optimization and particle stagnation when they reach the position boundary, a boundary rebound strategy was added.

The boundary bounce strategy is a strategy for limiting particles from exceeding the boundary of the search space. When the new position of the particle exceeds the boundary of the search space, in order to ensure the effectiveness and stability of the algorithm, this paper adopts the following strategy to bounce the particle’s position back into the search space.

Velocity boundary treatment:

(10) V=max(−vmax,min(vmax,V)),

Position boundary processing:

(11) X=max(xmin,min(xmax,X)),

(4) Steps for the damage quantitative detection method based on the IPSO algorithm

Step 1: Identify the general damage area according to the finite difference method, and determine the number of damaged units, unit numbers and other related parameters;

Step 2: Randomly generate the initial position and initial velocity of particles in the population;

Step 3: Calculate the fitness value of the particles, and record the individual optimal solution Pbest and the global optimal solution Gbest;

Step 4: Adaptively change ω,C1 and C2 (parameters related to the PSO algorithm);

Step 5: Update the particle speed and position according to the PSO algorithm;

Step 6: Find the speed boundary and position boundary;

Step 7: Add the MS operator to screen healthy units;

Step 8: Calculate the fitness value of the moved particles, and update Pbest and Gbest;

Step 9: Determine whether the maximum number of iterations Tmax is reached or whether the fitness value is zero; if it is not, then return to step 4;

Step 10: Output the current optimal particle (i.e., the optimization result), and terminate the algorithm.

## 3. Numerical Simulation Verification of Damage Detection Method

This section investigates the detection capability of the IPSO algorithm for various damage conditions, compares it with the PSO and GWO algorithms and analyzes the impact of noise on the performance of the IPSO algorithm.

### 3.1. Finite Element Model of Curved Laminated Plate

**Figure 2:**
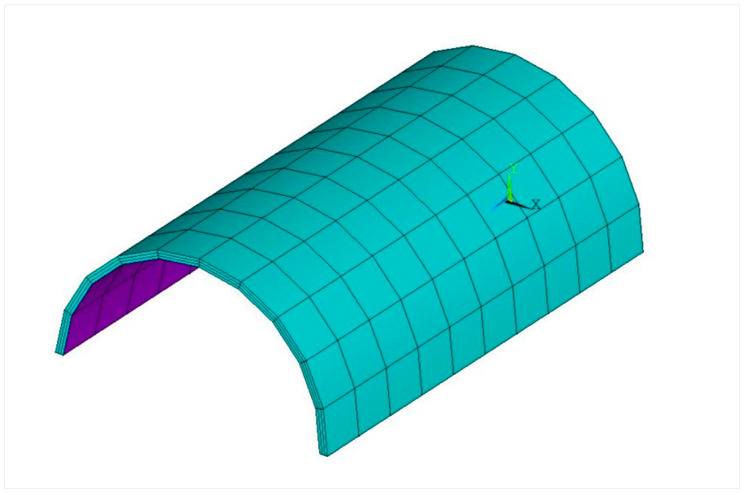
Numerical model of curved composite laminate.

In order to study the applicability of the proposed algorithm to the curved structure, this paper takes the curved composite laminates as the research object. The finite element model of the laminates was modeled and implemented using the ANSYS APDL parametric language, as shown in Figure 2. The size of the laminated plate was 0.35m×0.11πm×0.003m, and it was divided into three layers, with each layer being 0.001m, using carbon fiber composite material. The elastic modulus was $E_{1}\;=\;116\;\mathrm{GPa}$, $E_{2}\;=\;8.5\;\mathrm{GPa}$, $E_{3}\;=\;8.5\;\mathrm{GPa}$; Poisson’s ratio was $\mu_{12}\;=\;0.32$, $\mu_{13}\;=\;0.32$, $\mu_{23}\;=\;0.46$, and the density was $\rho \;=\;1800\;\mathrm{kg}/m^{3}$. The boundary condition of the laminated plate was fixed at both ends. The curved composite laminate was divided into 3 × 100 elements and 4 × 121 nodes using ANSYS software (Mechanical APDL 19.2).

**Figure 3:**
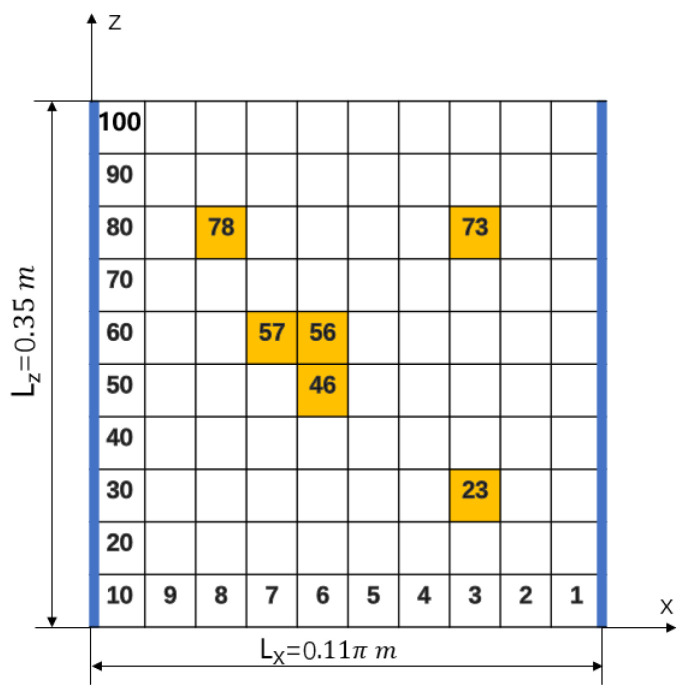
Damage unit number and location.

According to different damage conditions, four types of damage were set as shown in Table 1, and the location of the damaged unit is shown in Figure 3.

### 3.2. Detection Results of Different Damage Conditions

**Figure 4:**
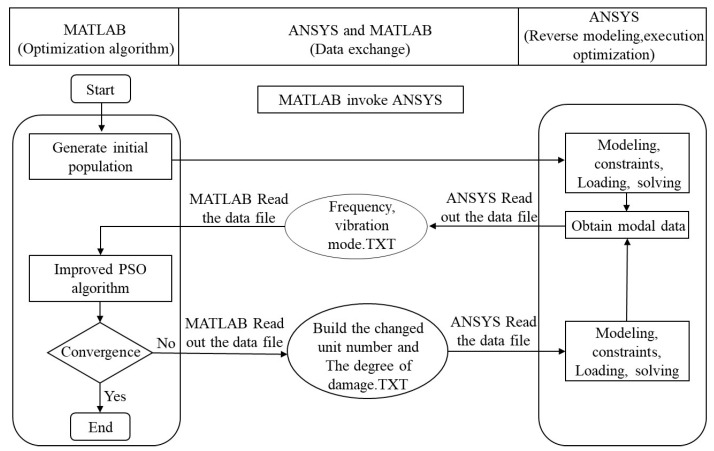
Inverse problem simulation platform based on MATLAB and ANSYS data interface.

The numerical simulation finite element model was established based on the ANSYS APDL parametric language, and IPSO was implemented through MATLAB software (MATLAB 2018a). The IPSO algorithm written in MATLAB served as the main program, and the finite element analysis was performed by having ANSYS as an auxiliary calculation in the main program. The calculation of relevant parameters was realized by reading and inputting the corresponding text through ANSYS and MATLAB. Taking the structural damage assessment method of curved composite laminates as an example, the calculation process of the inverse problem calculation simulation platform based on the MATLAB and ANSYS data interface [19,20] is shown in Figure 4 for the four types of damage conditions mentioned above.

#### Two Discrete Damage Locations

**Figure 5:**
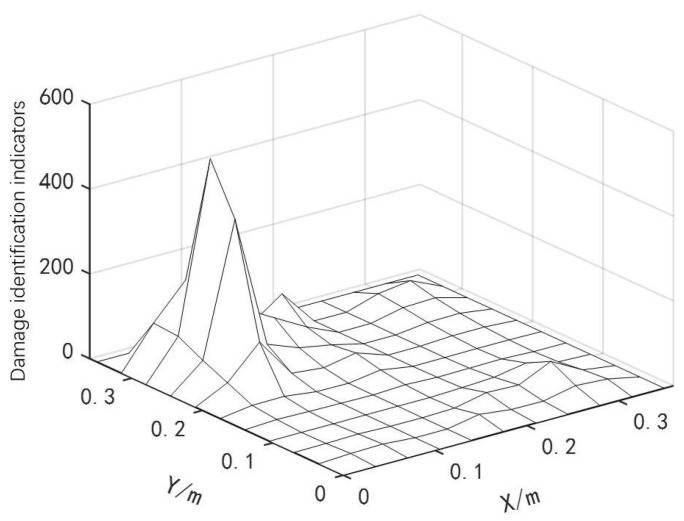
Location identification for two discrete damage types.

By using Equation (4), the modal curvatures of the damaged and healthy curved composite laminates in the x and y directions were obtained. These curvatures were then substituted into Equation (5), and a structural damage detection index was constructed using the differential method to evaluate the structural damage situation. The location identification results of the two discrete damage types are shown in Figure 5.

**Figure 6:**
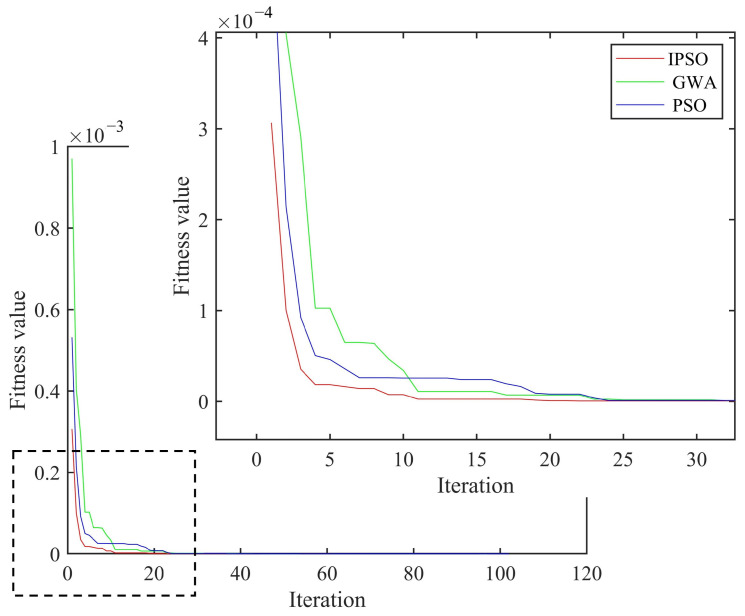
Average convergence curve of two discrete damage types.

As shown in Figure 5, the curved fiber-reinforced composites exhibited obvious bulges at the damage types (with more pronounced bulges in areas of severe damage and smaller bulges in areas of minor damage), allowing for preliminary determination of the damage positions and the identification of potential damaged element numbers: [22 23 24 77 78 79]. Based on the damage type recognition results from the finite difference method, combined with the IPSO, PSO and GWO algorithms, the damage positions and degrees of the curved fiber-reinforced composites could be quantitatively detected by invoking the computational data from MATLAB and ANSYS software. The convergence curve is presented in Figure 6, and the quantitative detection results for the two discrete damage types of the curved fiber-reinforced composites are listed in Table 2. Among the three algorithms, the IPSO algorithm achieved the lowest recognition result error rates and the fewest convergence iterations.

### 3.3. Two Consecutive Damages

**Figure 7:**
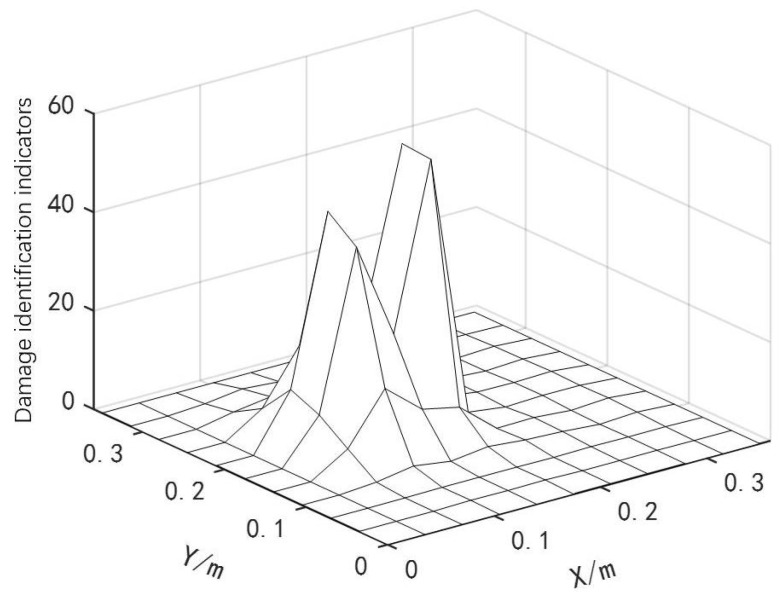
Location identification for two consecutive damage types.

The data processing method was the same as that in the previous section. The location identification results of two continuous damage types are shown in Figure 7.

**Figure 8:**
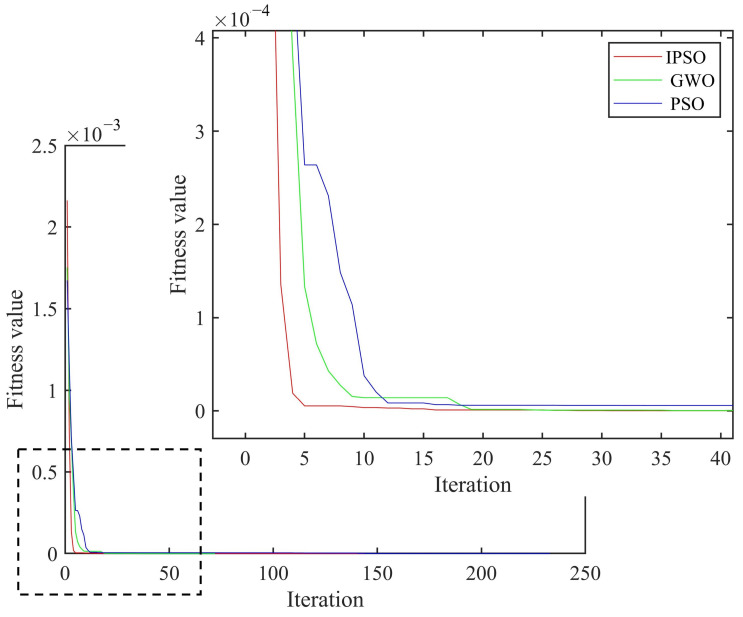
Average convergence curve of two consecutive damage types.

As shown in Figure 7, the curved fiber-reinforced composites exhibited obvious bulges at the damage locations, enabling preliminary determination of the damage positions and the identification of potential damaged element numbers: [46 47 48 56 57 58 66 67 68]. Based on the damage localization results from the finite difference method, and combined with the IPSO, PSO and GWO algorithms, the damage positions and degrees of the curved fiber-reinforced composites could be quantitatively detected. The convergence curves are presented in Figure 8, and the quantitative detection results for the two continuous damage types in the curved fiber-reinforced composites are listed in Table 3. Among the three algorithms, the IPSO algorithm achieved the lowest recognition error rates and the fewest convergence iterations.

#### 3.3.1. Three Damages

**Figure 9:**
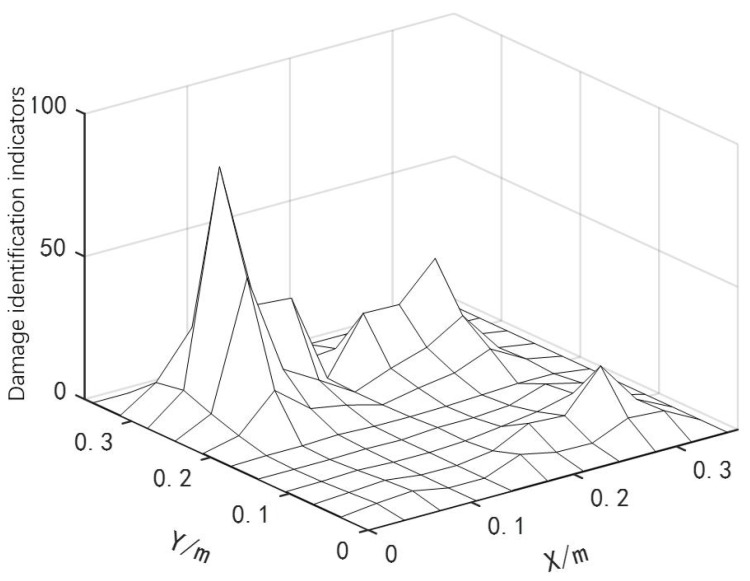
Location identification for three damage types.

The data processing method was the same as that in the previous section. The location identification results of the three damage types are shown in Figure 9.

**Figure 10:**
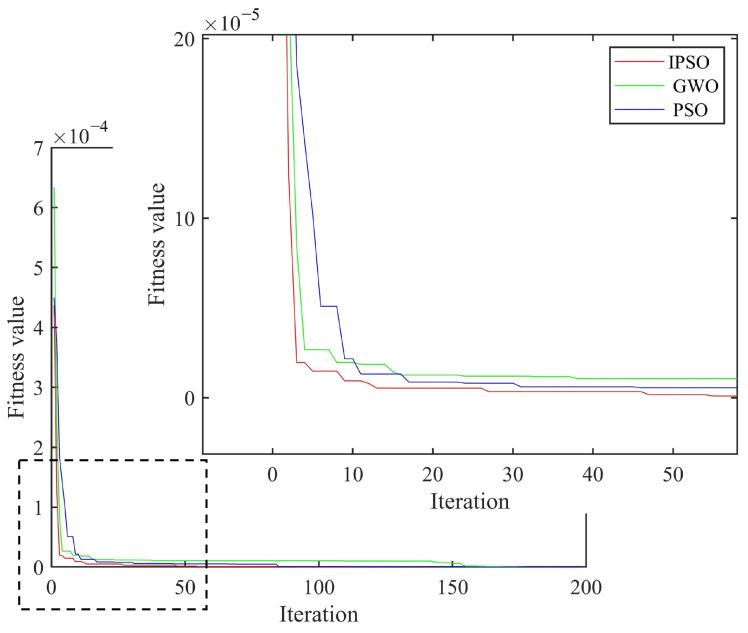
Average convergence curve of three consecutive damage types.

As shown in Figure 9, the curved fiber-reinforced composites exhibited obvious bulges at the damage locations (with more pronounced bulges in areas of greater damage and smaller bulges in areas of lesser damage), enabling preliminary determination of the damage positions and identification of the potential damaged element numbers: [22 23 24 72 73 74 77 78 79]. Based on the damage localization results from the finite difference method, and combined with the IPSO, PSO and GWO algorithms, the damage positions and degrees of the curved fiber-reinforced composites can be quantitatively detected. The convergence curves are presented in Figure 10, and the quantitative detection results for the two continuous damage types in the curved fiber-reinforced composites are listed in Table 4. Among the three algorithms, the IPSO algorithm achieved the lowest recognition error rates and the fewest convergence iterations. The table shows that the number of convergence iterations for the algorithm when detecting three damage types was significantly higher than when detecting two damage types.

#### 3.3.2. Four Damages

**Figure 11:**
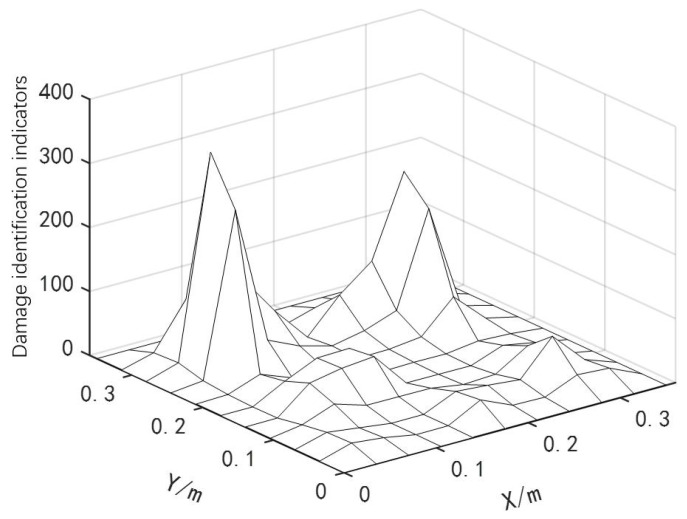
Location identification for four damage types.

The data processing method was the same as that in the previous section. The location identification results of the four damage types are shown in Figure 11.

**Figure 12:**
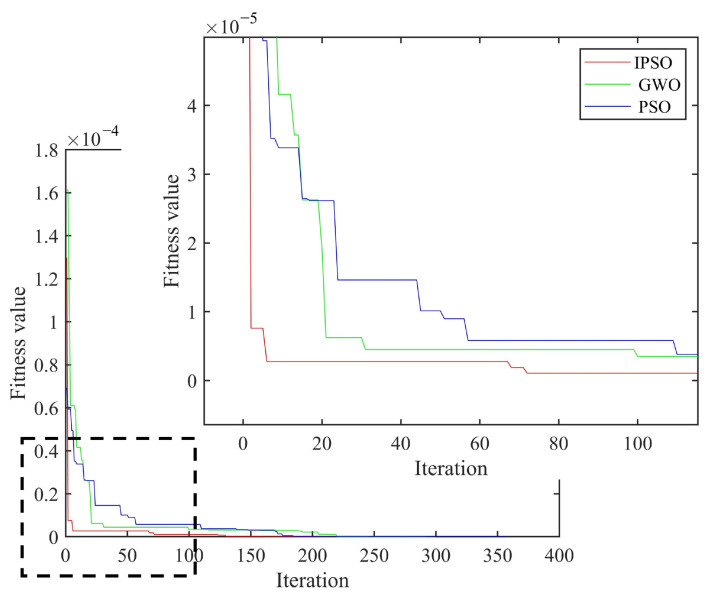
Average convergence curve of four damage types.

As shown in Figure 11, the numbers of the potential damaged elements were identified: [22 23 24 45 46 47 72 73 74 77 78 79]. Based on the damage localization results from the finite difference method, and combined with the IPSO, PSO, and GWO algorithms, the damage positions and degrees of the curved fiber-reinforced composites could be quantitatively detected. The average convergence curves are presented in Figure 12, and the quantitative detection results for the four continuous damage types in the curved fiber-reinforced composites are listed in Table 5. Among the three algorithms, the IPSO algorithm achieved the lowest recognition error rates and the fewest convergence iterations. The table shows that the number of convergence iterations for the algorithm when detecting four damage types was significantly higher than when detecting two or three damage types.

As shown by the detection results of the four types of damage, the results based on the finite difference method could accurately identify the approximate area and number of damage types. The numbers of damaged elements identified by the IPSO algorithm were consistent with the preset values, and the error rates in the damage degree of each element were kept below 0.5%. Compared with the detection results of the PSO and GWO algorithms, the IPSO algorithm exhibited lower error rates and fewer convergence iterations, effectively verifying the reliability of the proposed algorithm. However, as the complexity of the damage conditions increased, the computational load required by the IPSO algorithm rose significantly, leading to a substantial increase in both the number of convergences and the corresponding detection time.

### 3.4. Analysis of Damage Detection Ability of IPSO Algorithm

#### 3.4.1. The Influence of the Damage Degree on the Detection Ability of the Algorithm

In order to further evaluate the detection ability of the algorithm, this section studies the detection effect of the algorithm on different degrees of damage. Taking the No. 56 damage unit as an example, the damage degree of the unit was set to 5%, 10%, 70% and 95%. In order to verify the accuracy of the proposed method for large damage and small damage detection, the quantitative damage detection results are shown in Table 6.

According to the detection results, regardless of the size of the damage, the proposed algorithm could effectively detect the damage of the unit. However, if the unit was set as small damage, then the detection error would be relatively larger compared with large damage, indicating that the algorithm has a slightly stronger detection ability for large damage than for small damage.

#### 3.4.2. The Influence of Noise on the Detection Ability of the Algorithm

In the actual modal test, due to the constant presence of measurement noise or errors, both the frequency and mode shapes would be polluted by noise, which put forward certain requirements for the anti-noise ability of the algorithm. Therefore, this section studies the influence of noise on the performance of the IPSO algorithm. The noise added to the frequency and mode shapes was as follows:

(12) $$\omega_{i}^{\mathrm{noise}\;}=\left(1+\eta_{f}\left(2\mathrm{rand}\left[0,1\right]-1\right)\right)\omega_{i}$$

(13) $$\psi_{ij}^{\mathrm{noise}\;}=\left(1+\eta_{\psi }\left(2\mathrm{rand}\left[0,1\right]-1\right)\right)\psi_{ij}$$

In the formula, $\omega_{i}^{\mathrm{noise}\;}$ is the *i*th natural frequency, $\psi_{ij}^{\mathrm{noise}\;}$ is the *j*th component of the *i*th mode shape vector, and η is the noise level.

**Figure 13:**
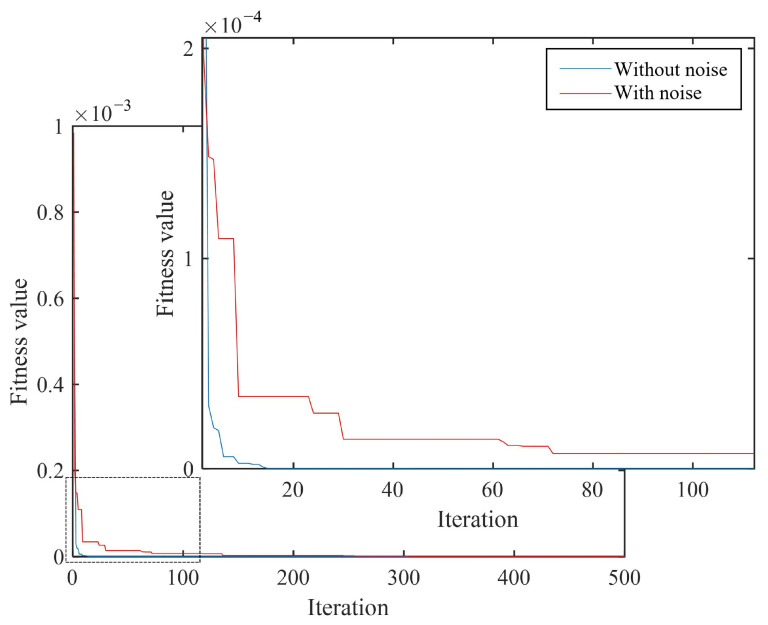
Comparison of convergence curves of two discrete damage types.

**Figure 14:**
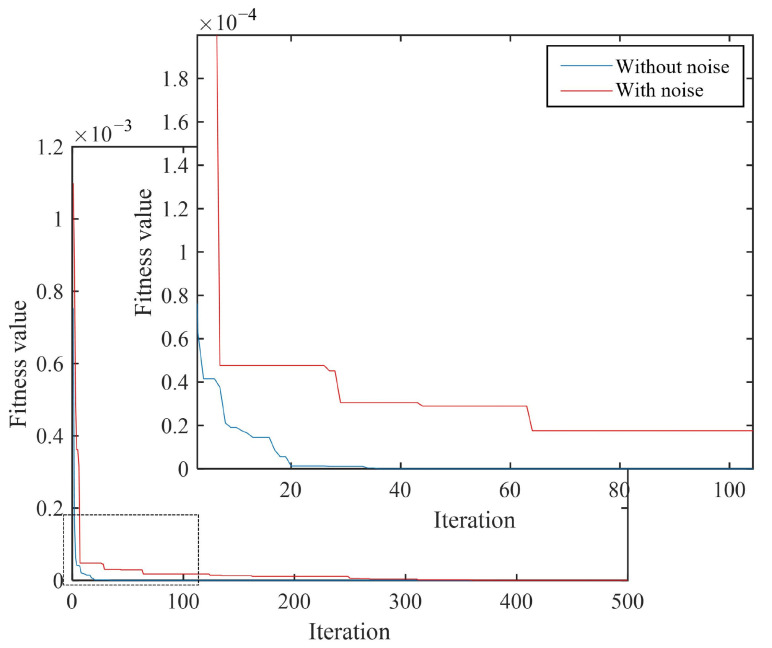
Comparison of convergence curves of two consecutive damage noises.

In the following examples, in order to study the robustness of the proposed method, a relatively high noise level was added to the structural frequency and mode, where the frequency error $\eta_{f}=1\%$ and the vibration mode error $\eta_{\psi }=5\%$. Numerical simulations were performed on two discrete and two continuous damage types. The test results are shown in Table 7 and Table 8 and Figure 13 and Figure 14.

According to the quantitative damage detection results in Table 7 and Table 8, it can be seen that under the condition of no noise, the detection error rates of both types of damage conditions were below 0.3%. After adding noise interference (frequency and mode noise were 1% and 5%, respectively), the IPSO algorithm could still accurately detect the damage degree in both types of damage conditions. The detection error rates of two discrete damage types were 4.31% and 2.54% respectively, while the detection error rates were 5.70% and 5.40%, respectively, which were much larger than those without noise. From Figure 13 and Figure 14, it can be seen that under the two types of damage conditions, the convergence speed of the algorithm slowed down, and the number of convergence interations increased significantly after adding noise.

## 4. Experimental Verification

**Figure 15:**
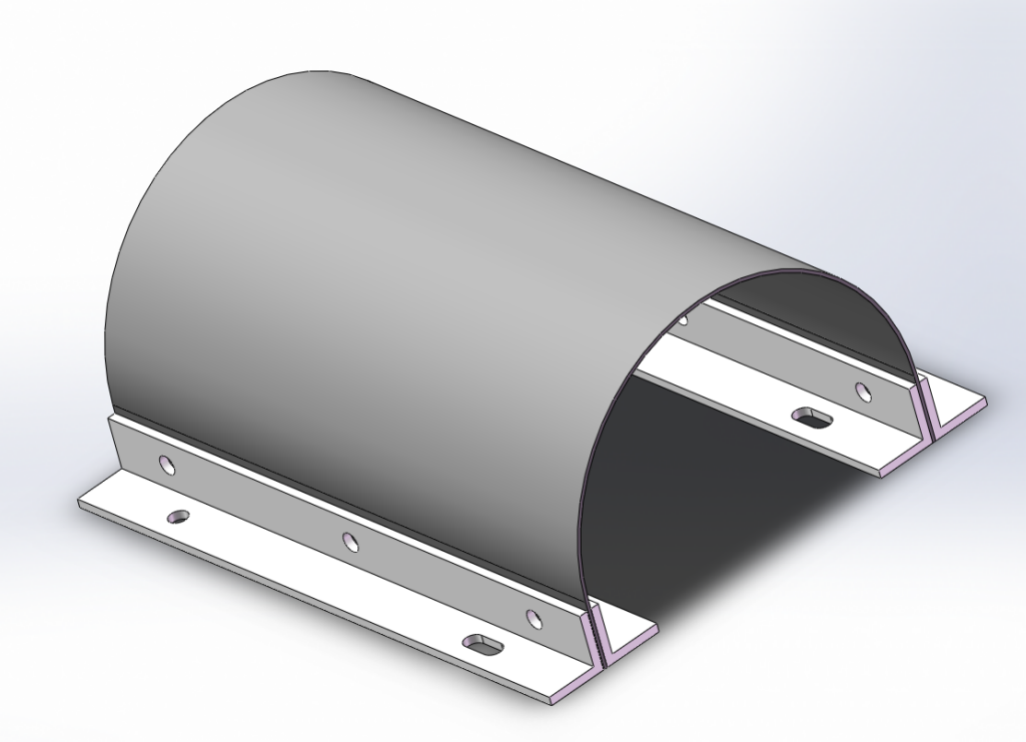
Schematic diagram of test specimen.

In this paper, an experiment was performed to verify the feasibility of the IPSO algorithm for structural damage assessment of curved structures. As a verification experiment, in order to reduce the testing error caused by the processing error of the experimental specimens, steel plates with a simpler structure and material parameters were selected instead of carbon fiber curved laminates as the experimental test specimens. The three-dimensional modeling diagram of the experimental specimens is shown in Figure 15. The size of the specimen was 0.36 m × 0.11 π m × 0.001 m, with a plate thickness of 0.001 m. A clamping allowance of 0.005 m was reserved at both ends of the specimen.

**Figure 16:**
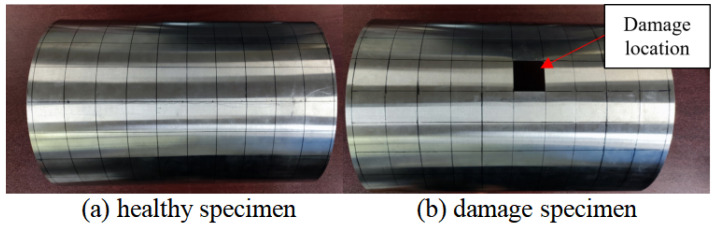
Test specimen.

Before starting the experimental testing, it was necessary to divide the experimental specimen into a grid to determine the excitation and detection positions. The structure was evenly divided into 100 units, with unit sizes of 0.036 m × 0.035 m and a total of 121 nodes. The damage was set to a designated location, with unit number 56 and a damage degree of 100%. The healthy and damaged specimens are shown in Figure 16a,b, respectively.

### 4.1. Test System

**Figure 17:**
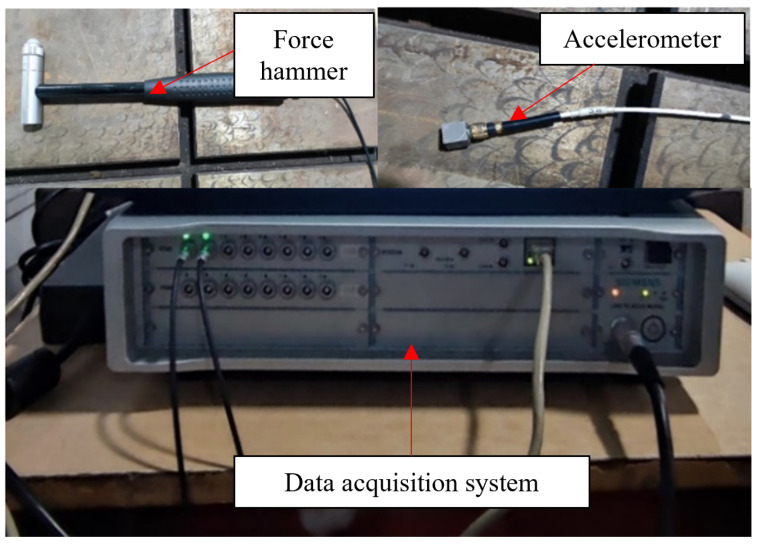
Main experimental instrument.

The experimental test software system adapted the modal analysis module of SIEMENS LMS Test Lab 18A. The experimental instruments mainly included an LMS SCADAS310 multi-function data acquisition system (LMS Instruments, Leuven, Belgium), unidirectional ICP accelerometer, force hammer, computer and fixture. The instruments used in the vibration experiment are shown in Figure 17.

**Figure 18:**
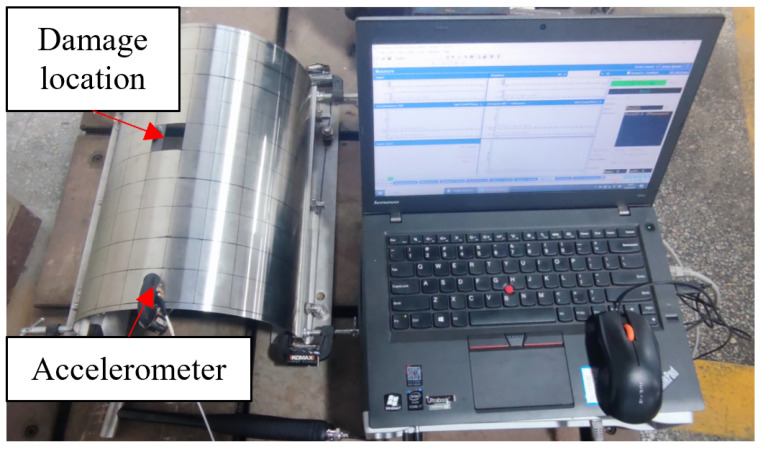
Vibration testing system.

The test specimen was fixed on the test bench with vises and bolts. According to the relevant experimental conditions, the vibration test experimental system is shown in Figure 18.

The steps for measuring and collecting the experimental signals were as follows:

(1) Firstly, it was confirmed that the instruments and equipment used in the experiment met the experimental requirements, and the necessary functional inspection and calibration work were completed;

(2) The test specimen was firmly installed on the experimental platform through the fixtures, ensuring that the test specimen was in a stable and non-loose state throughout the entire experiment period;

(3) Whether the wiring was correct was checked, as was whether the indicator light of the data acquisition instrument was normal;

(4) The corresponding structural models for the tested objects were established in the experimental testing system, debug key component parameters such as the accelerometers and force hammers and completing the experimental parameter settings;

(5) During the experiment, the measurement strategies of multi-point excitation and single-point detection were adopted. According to the pre-planned grid node order, the force hammer was moved one by one for tapping excitation, and the structural vibration response data under different position excitations were collected;

(6) Finally, all of the frequency response function data obtained during the experiment were summarized, and the natural modal frequency and corresponding modal shape of the structure were solved using the Time MDOF module for comprehensive calculation and processing so as to carry out subsequent damage detection and analysis.

### 4.2. Analysis of Experimental Measurement Signal and Processing Results

The hammer was moved one by one to complete the knocking test for 121 nodes of the experimental specimen. Each node was knocked three times to find its average value. Then, the first five natural frequencies of the experimental specimen could be obtained with LMS TEST LAB software (LMS Test Lab 2021) and compared with the theoretical natural frequencies obtainted from numerical simulation. The results are shown in Table 9.

**Figure 19:**
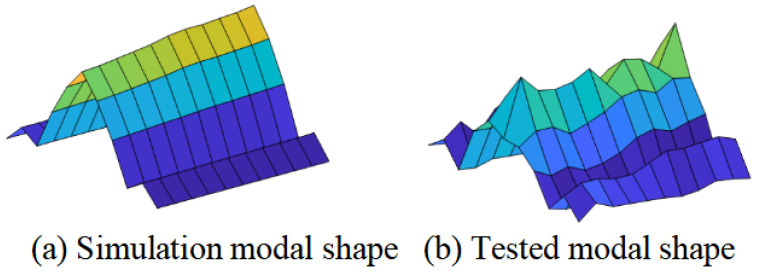
The second-order modal shapes of the specimen.

Due to the relatively large error rate in the first-order frequency measured in the experiment and the fact that the modal shapes corresponding to the low-order modes were more conducive to structural damage assessment, the modal parameters corresponding to the second-order frequency were selected for experimental verification. The second-order modal shapes of healthy structures obtained from numerical simulation and experimental testing are shown in Figure 19a,b, respectively. Regardless of the noise factors, the two were basically consistent.

**Figure 20:**
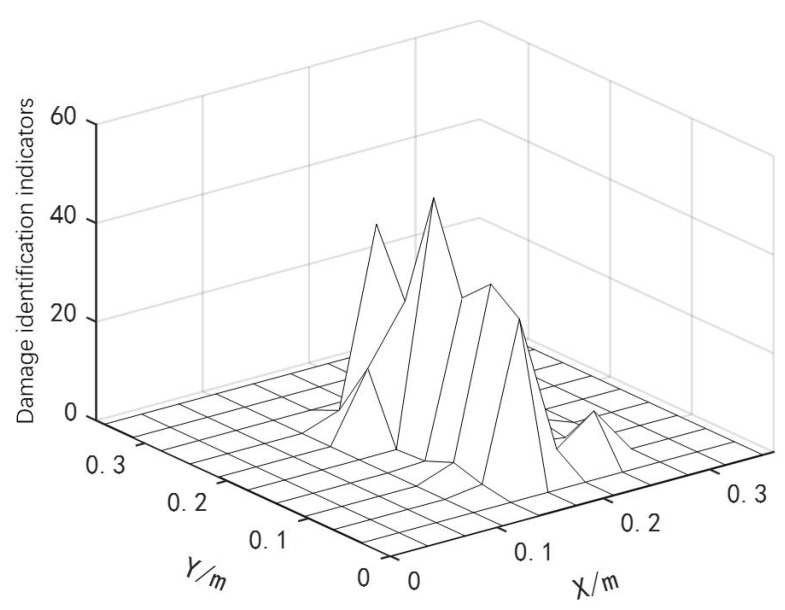
Experimental results of structural damage assessment based on differential method.

The obtained second-order modal shapes were substituted into Equation (4) to calculate the curvature of the modal shapes before and after structural damage. Based on the differential method described in Equation (5), a structural damage assessment index was constructed, and the structural damage assessment results are shown in Figure 20.

From Figure 20, it can be identified that the damage units within the damaged area were numbered as [45 46 47 55 56 57 65 66 67]. These data were input into the damage quantitative detection method based on the IPSO algorithm. After normalization, optimization was performed on individual data with larger error rates. Each set of damage conditions was run three times, and the damage quantitative detection results are shown in Table 10.

From the quantitative damage detection results in Table 10, it can be seen that the damage detection method based on the IPSO could accurately identify the location of the damage unit and accurately quantify the damage degree of the specimen. The damage degree detected by the three operation results was not significantly different, which also proves the strong robustness of the method and verifies the feasibility of the method proposed in the numerical analysis.

## 5. Conclusions

A quantitative damage detection algorithm based on IPSO was proposed for structural damage assessment of curved layered composite structures. Firstly, based on the differential method, the approximate location of the damage was identified, and then an IPSO algorithm was used to perform location recognition and quantitative calculation. The effectiveness, robustness and scalability of the proposed method were verified through numerical examples of four damage conditions in curved composite laminates. In addition, the damage detection ability of the algorithm was further validated by setting small and large damage types, and the influence of noise on the detection performance of the algorithm was also studied. Finally, the proposed algorithm was validated through experiments. Although the noise problem during the experiment may have had a certain impact on the measured data and structural damage assessment results, the method proposed in this paper can still detect the location of damage and quantify the degree of damage, further verifying the feasibility of the IPSO algorithm for structural damage assessment of layered composite materials with curved configurations. Based on the analysis results, the following conclusions can be drawn:

(1) By using the differential method to identify the damaged areas of coposit laminates and find a set of potential damaged elements, the computational cost of the IPSO algorithm can be effectively reduced. The developed objective function can effectively identify the location and degree of damage to the damaged units without any false alarms. As the number of damage types and complexity of the damage type settings increase, the convergence times also increase.

(2) To prevent stagnation when particles reach the position boundary, a boundary rebound strategy can be added for boundary processing. At the same time, introduction of the MS operator greatly accelerates the convergence speed of the algorithm and reduces the influence of non-damaged elements on the actual damage degree of damaged elements.

(3) The proposed two-stage damage detection method is an effective optimization tool. The IPSO algorithm can not only quantify the damage degree correctly in the absence of noise but also in polluted noise data (frequency and mode noise of 1% and 5%, respectively) with detection errors within 6%.

For future research, the IPSO damage detection algorithm is expected to solve the problems of multivariate and accelerated convergence, be gradually expended to solve the problem of complex irregular surface damage detection and then applied to solve practical engineering detection problems.

## Figures and Tables

**Table 1:** Four types of damage conditions.

Num.	Damage Condition	Number of Damage Unit	Damage Degree
1	Two discrete damage types	23/78	30%/70%
2	Two consecutive damage types	56/57	30%/30%
3	Three damage types	23/73/78	20%/30%/40%
4	Four damage types	23/46/73/78	30%/60%/60%/70%

**Table 2:** Quantitative detection results of two discrete damage types (23/78).

Algorithm Name	Preset Value	Detected Value	Number of Convergences	Error (%)
PSO	0.3/0.7	0.3040/0.7036	102	1.33/0.51
GWO	0.3/0.7	0.3030/0.6960	97	1.0/0.57
IPSO	0.3/0.7	0.3005/0.6993	60	0.17/0.10

**Table 3:** Quantitative detection results of two consecutive damage types (56/57).

Algorithm Name	Preset Value	Detected Value	Number of Convergences	Error (%)
PSO	0.3/0.3	0.295/0.297	151	1.6/1.0
GWO	0.3/0.3	0.296/0.297	144	1.3/1.0
IPSO	0.3/0.3	0.2985/0.2994	93	0.50/0.20

**Table 4:** Quantitative detection results of three damage types (23/73/78).

Algorithm Name	Preset Value	Detected Value	Number of Convergences	Error (%)
PSO	0.2/0.3	0.196/0.311	198	2.0/3.33
	/0.4	/0.413		/3.25
GWO	0.2/0.3	0.203/0.303	188	1.5/1.0
	/0.4	/0.412		/3
IPSO	0.2/0.3	0.196/0.3012	149	0.15/0.27
	/0.4	/0.4014		/0.37

**Table 5:** Quantitative detection results of four damage types (23/46/73/78).

Algorithm Name	Preset Value	Detected Value	Number of Convergences	Error (%)
PSO	0.3/0.6	0.305/0.611	357	1.66/1.83
	/0.6/0.7	0.591/0.689		1.5/1.57
GWO	0.3/0.6	0.296/0.589	305	1.3/1.83
	/0.6/0.7	0.604/0.703		0.66/0.42
IPSO	0.3/0.6	0.3014/0.6004	226	0.46/0.06
	/0.6/0.7	0.6002/0.7012		0.03/0.17

**Table 6:** Quantitative testing results for unit 56.

Damage Degree	5%	10%	70%	95%
Damage unit number	56	56	56	56
Optimal solution	0.0523	0.1028	0.7009	0.9494
Average solution	0.0510	0.1014	0.6999	0.9517
Standard deviation	0.0027	0.0030	0.0037	0.0023
Error (%)	2.00	1.40	0.01	0.18

**Table 7:** Quantitative detection results of two discrete damage types.

	Without Noise		With Noise	
Damage unit number	23	78	23	78
Optimal solution	0.3005	0.7003	0.3059	0.6914
Average solution	0.3005	0.6993	0.2870	0.6822
Standard deviation	0.0024	0.0013	0.0151	0.0160
Error (%)	0.17	0.10	4.31	2.54

**Table 8:** Quantitative detection results of two consecutive damage types.

	Without Noise		With Noise	
Damage unit number	56	57	56	57
Optimal solution	0.2985	0.2994	0.2807	0.3039
Average solution	0.2994	0.2994	0.3171	0.2838
Standard deviation	0.0033	0.0014	0.0324	0.0164
Error (%)	0.20	0.20	5.70	5.40

**Table 9:** Comparison of simulation and experimental results of the first five natural frequencies.

Modal Order	Simulation Frequency (Hz)	Experimental Frequency (Hz)	Relative Error (%)
1	88.561	76.433	13.69
2	194.39	185.432	4.61
3	198.21	189.377	4.46
4	327.33	341.807	4.42
5	360.31	354.737	1.55

**Table 10:** Experimental results of quantitative damage detection.

	The First Run Results	The Second Run Results	The Third Run Results
Damage unit number	56	56	56
Damage degree	0.9836	0.9765	0.9890

## Data Availability

The original contributions presented in this study are included in the article. Further inquiries can be directed to the corresponding author.
